# Angle closure secondary to lens remnants in a patient with presumed aphakia: case report

**DOI:** 10.1186/s12886-022-02338-6

**Published:** 2022-04-05

**Authors:** Kristi Y. Wu, Lance J. Lyons, Gavin W. Roddy

**Affiliations:** Mayo Clinic Department of Ophthalmology, 200 1st Street SW, Rochester, MN 55905 USA

**Keywords:** Angle closure, Aphakia, Congenital cataract, Case report

## Abstract

**Background:**

Eyes with a short axial length or anterior chamber depth often develop narrowed anterior chamber angles in association with an enlarging crystalline lens. We report a case of a patient who presented in angle closure, with a distant history of prior intervention for congenital cataracts and was presumed to be aphakic.

**Case presentation:**

A 78-year-old male presented with acute onset unilateral eye pain and blurred vision. He was found to have increased intraocular pressure, anteriorly bowed iris, and angle closure. Despite prior documentation of aphakia after treatment for congenital cataracts, detailed workup revealed residual crystalline lens material pushing the peripheral iris anteriorly. Further history confirmed that the patient underwent a procedure in the 1940’s to remove lens material centrally but was not truly aphakic. The patient was treated with anterior chamber paracentesis and intraocular pressure lowering drops. His intraocular pressure remains controlled with medical therapy alone.

**Conclusions:**

Patients that appear to be aphakic centrally may still present with angle closure secondary to residual peripheral lens material. This case highlights the importance of keeping this etiology on the differential in a patient with presumed aphakia.

## Background

In the last century, the surgical approach to congenital cataracts has greatly evolved and progressed with the advancement of technological innovation, instrumentation, and surgical approach [1]. We present the case of a patient with a long-term complication of a prior congenital cataract surgical intervention and how it may be distinguished from other conditions.

## Case presentation

**Fig. 1:**
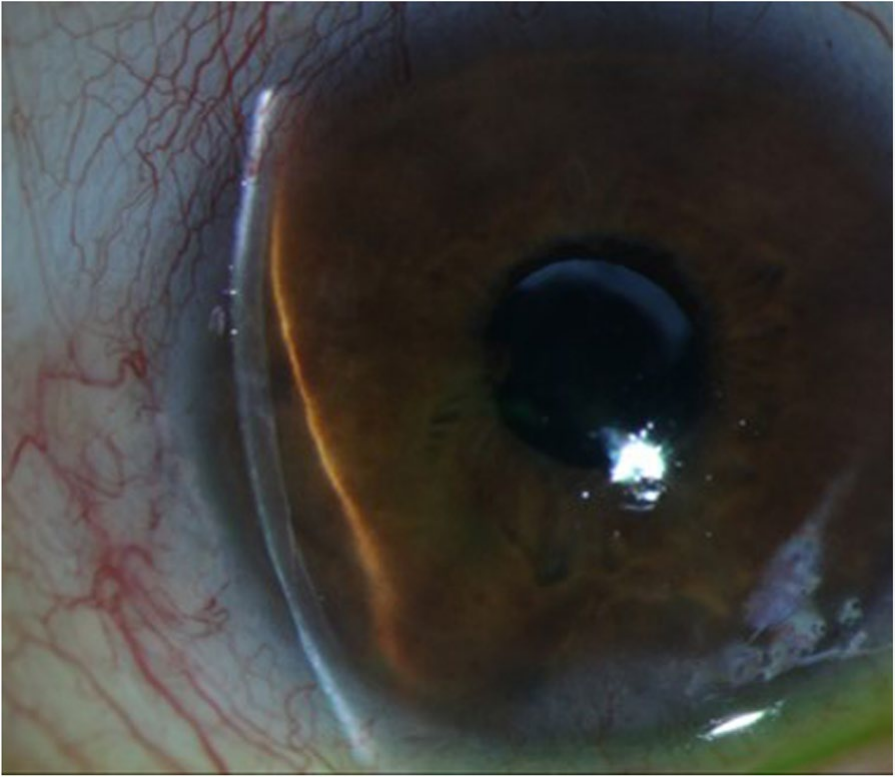
*Slit lamp image of the right eye.* The temporally placed slit beam demonstrates narrowing of the superior angle and anterior bowing of the superior iris. There is no lens visible through the pupil

**Fig. 2:**
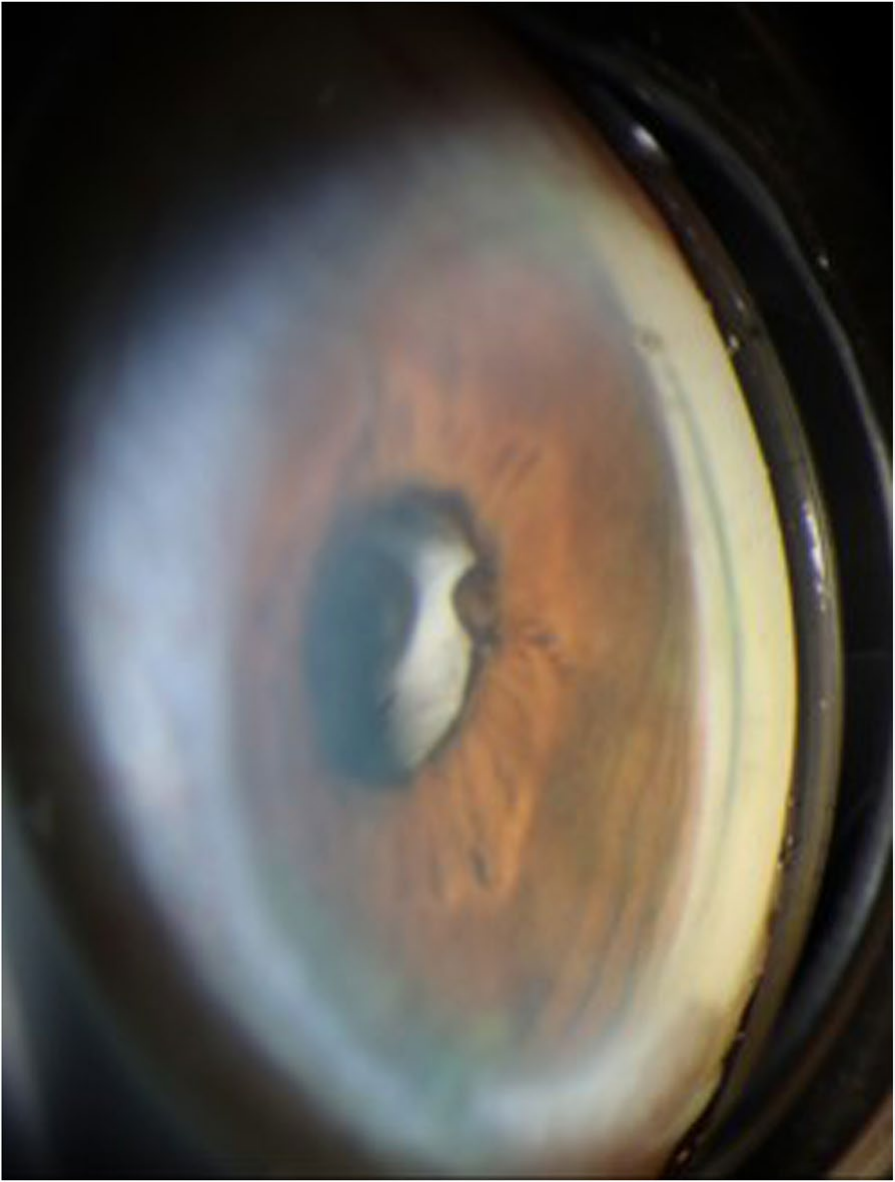
*Gonioscopic image of the right temporal angle.* The iris is bowed forward with no trabecular meshwork visible, superotemporally in contrast to the adjacent inferior angle, which is open to ciliary body. An irregular white mass is visible posterior to the iris

**Fig. 3:**
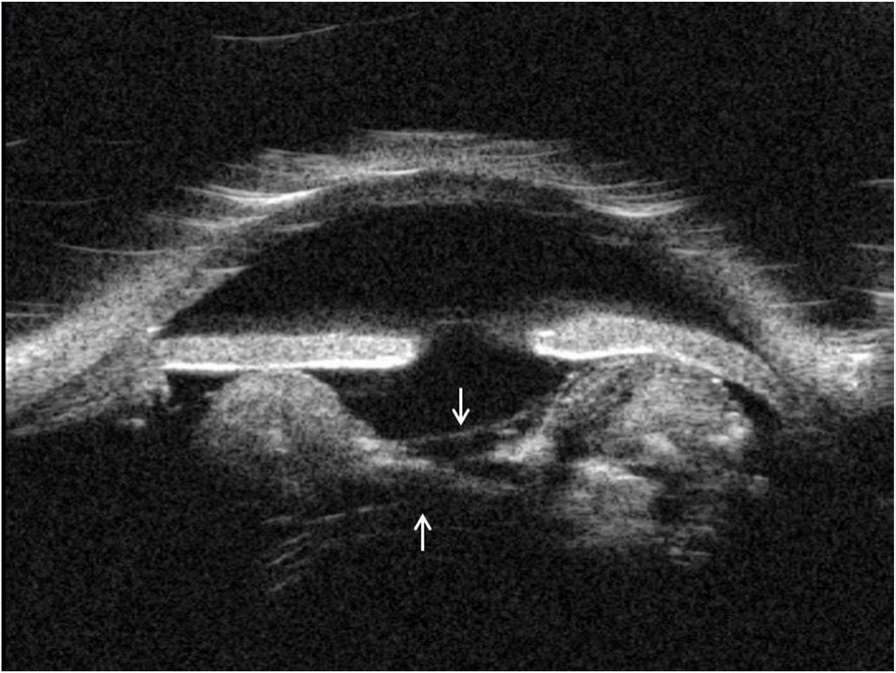
*Ultrasound biomicroscopy of the right eye.* This is a vertical axial cut that shows a narrowed superior angle, on the right in the image. There is a heterogeneously echogenic mass posterior to the iris that is causing anterior bowing of the superior iris. The anterior and posterior capsules appear intact, as indicated by arrows

A 78-year-old male presented with 3 days of intermittent blurred vision and pain of the right eye (OD). His medical history included Crohn’s colitis, Addison’s disease, and chronic obstructive pulmonary disease. By chart review, his ocular history included aphakia in both eyes (OU) following intervention for congenital cataracts during childhood in the 1940’s, microcornea OU, aphakic glaucoma OD, and no light perception vision in the left eye (OS) secondary to vision loss following a congenital cataract surgical procedure at age 18, currently with an opaque and vascularized cornea. Although axial length and anterior chamber depth have not been measured OU, the patient is known to have a macular coloboma OD with subjective refraction of − 1.00. Intraocular pressure (IOP) was previously well-controlled in the low teens OU on daily latanoprost 0.005% OD, and central corneal thickness was 549 μm OD and 648 μm OS. On presentation, Snellen visual acuity OD was 20/200 at baseline. The IOP by Goldmann applanation tonometry was elevated to 37 mmHg OD. Slit lamp examination revealed anterior bowing of the superotemporal iris with resulting narrowed angle (Fig. 1). Gonioscopy from 9:00–12:00 demonstrated iris apposition to the trabecular meshwork with an irregular, white globular mass posterior the iris (Fig. 2). There was no evidence of peripheral anterior synechiae, and the optic nerve appeared stable with cup to disc ratio of 0.8. Ultrasound biomicroscopy (UBM) confirmed that there was a hyperechoic circumferential mass causing anterior bowing of the superotemporal iris and angle closure (Fig. 3). Furthermore, adjacent to the presumed crystalline lens remnants on UBM, apparent anterior and posterior lens capsule were visualized (Fig. 3 arrows). The patient was treated with topical and oral aqueous suppressive medications, without improvement in IOP. Due to the patient’s history of pulmonary disease and adrenal insufficiency, beta blockers and hyperosmotic agents were not administered. Subsequently, the patient had a paracentesis of the anterior chamber with IOP decreased to 8 mmHg. The patient was continued on topical latanoprost 0.005% nightly, brimonidine 0.2% 3 times daily, and dorzolamide 2% 3 times daily. Subsequent manual visual field testing did demonstrate progressive field loss, and the patient was offered more definitive intervention including laser peripheral iridotomy (LPI) or surgical removal of lens remnants. The patient declined intervention and remains on medical management with close follow-up, with the understanding that symptomatic recurrence of angle closure would likely necessitate further intervention.

## Discussion and conclusions

Our patient has a history of congenital cataracts OU, for which he underwent surgical intervention in the 1940’s. He described a “needling procedure” OD, rather than removal of the complete lens or lens-capsule complex. The hospital where this procedure was performed had been closed, and medical records were not available. However, findings on slit lamp examination and UBM support this history. Gonioscopy showed a circumferential opalescent mass posterior to iris that was present 360 degrees, but most prominent in the superotemporal quadrant. UBM demonstrated intact anterior and posterior lens capsules, with a round, echogenic mass inside the lens capsule. Together, these findings suggested residual crystalline lens, rather than Soemmering’s ring, as the underlying cause of angle closure. The increase in IOP, however, was likely multifactorial, as appositional closure was noted for less than 180 degrees, and this patient already had known risk factors for glaucoma including prior surgery for congenital cataracts and microcornea [2].

With the modern-day development of vitrectomy and phacoemulsification units, this amount of residual crystalline lens would be atypical. In the 1940’s, however, this technology was not available, so intervention for congenital cataracts included discission and linear extraction [1]. Discission involves puncturing the anterior lens capsule and subsequent absorption of lens material into the aqueous, while linear extraction requires a larger corneal incision with expression of lens and aqueous through the wound. Both procedures aim to clear the visual axis and minimize amblyopia. This patient’s description of a “needling” procedure to disrupt the central lens, as well as our examination findings, support this intervention in our patient.

It is important to recognize residual crystalline lens as a potential cause of secondary angle closure, even in a patient with history of aphakia. This differential diagnosis of similar presentations ﻿includes lens material, iris mass, ciliary body mass, and choroidal effusion. Two reported cases describe patients with prior intervention for congenital cataracts that years later, were referred for concern for iris lesions [3, 4]. One of the patients had a narrowed angle and elevated IOP similar to our patient, while the other patient was asymptomatic. Both patients had UBM imaging that confirmed localization of the lesion to residual crystalline lens, and they were medically managed. Aphakic eyes are also at risk of developing peripheral anterior synechiae and chronic angle closure, especially in the setting of complications such as pupillary block from the vitreous face and choroidal effusion [5]. The chronic angle closure may require more surgical intervention, which would be contraindicated in cases of primary iris or ciliary body neoplasm.

This phenomenon of angle closure in aphakic patients has an analogous presentation in pseudophakic patients. A series of 3 patients in angle closure due to a Soemmering’s ring were successfully treated with LPI [6]. Another patient had plateau iris and a Soemmering’s ring that improved with topical pilocarpine [7]. Similar to the cases of aphakic patients, it is the slow proliferation of lens epithelial cells that results in mechanical displacement of the iris and obstruction of the trabecular meshwork. In another case, angle closure occurred in a non-pupillary block mechanism, as evidenced by a patient where LPI did not lead to resolution of closure [8]. Fortunately for these patients, most respond adequately to medical treatment with the correct diagnosis.

In summary, we present a case of a 78-old-male that presented with angle closure secondary to lens remnants, even after prior intervention for congenital cataracts. Although our patient strongly wished to avoid any medical procedures, both LPI and extraction of lens remnants were considered. This case highlights the importance of conducting thorough history, exam, and imaging in all patients, even those with prior ocular diagnoses.

## Data Availability

Not applicable.

## Abbreviations

OD: Right eye
OU: Both eyes
OS: Left eye
IOP: Intraocular pressure
UBM: Ultrasound biomicroscopy
LPI: Laser peripheral iridotomy
